# Self-assembly of metallosupramolecular rhombi from chiral concave 9,9’-spirobifluorene-derived bis(pyridine) ligands

**DOI:** 10.3762/bjoc.10.40

**Published:** 2014-02-18

**Authors:** Rainer Hovorka, Sophie Hytteballe, Torsten Piehler, Georg Meyer-Eppler, Filip Topić, Kari Rissanen, Marianne Engeser, Arne Lützen

**Affiliations:** 1 University of Bonn, Kekulé-Institute of Organic Chemistry and Biochemistry, Gerhard-Domagk-Str. 1, D-53121 Bonn, Germany; 2 Department of Chemistry, Nanoscience Center, University of Jyväskylä, P.O. Box 35, 40014 Jyväskylä, Finland

**Keywords:** concave templates, metal complexes, self-assembly, self-sorting, 9,9’-spirobifluorene, supramolecular chemistry

## Abstract

Two new 9,9’-spirobifluorene-based bis(4-pyridines) were synthesised in enantiopure and one also in racemic form. These ligands act as concave templates and form metallosupramolecular [(dppp)_2_M_2_L_2_] rhombi with *cis*-protected [(dppp)Pd]^2+^ and [(dppp)Pt]^2+^ ions. The self-assembly process of the racemic ligand preferably occurs in a narcissistic self-recognising manner. Hence, a mixture of all three possible stereoisomers [(dppp)_2_M_2_{(*R*)-L}_2_](OTf)_4_, [(dppp)_2_M_2_{(*S*)-L}_2_](OTf)_4_, and [(dppp)_2_M_2_{(*R*)-L}{(*S*)-L}](OTf)_4_ was obtained in an approximate 1.5:1.5:1 ratio which corresponds to an amplification of the homochiral assemblies by a factor of approximately three as evidenced by NMR spectroscopy and mass spectrometry. The racemic homochiral assemblies could also be characterised by single crystal X-ray diffraction.

## Introduction

Concave templates are versatile tools to achieve self-sorting behaviour in the self-assembly of defined aggregates from multicomponent mixtures in a social self-discrimination or a narcissistic self-recognition manner [1–3]. Usually, geometrical size and shape complementarity are employed to ensure such self-sorting. However, these factors do not account in self-sorting processes where enantiomers are involved whose shape and size does not vary but only their relative spatial orientation. Hence, chiral self-sorting is a truly challenging task and high-fidelity chiral self-sorting processes have been observed in a rather limited number of examples for the formation of stereochemically defined metallosupramolecular aggregates from racemic ligands in a self-recognising [4–18] or a self-discriminating manner [19–27].

As part of our ongoing programme to develop general guidelines for the diastereoselective self-assembly of metallosupramolecular aggregates, we have recently started a systematic study on the influence of rigid concave building blocks on the outcome of the self-assembly of bis(pyridine) ligands into dinuclear metallosupramolecular rhombi upon coordination to *cis*-protected [(dppp)Pd(OTf)_2_] or [(dppp)Pt(OTf)_2_] (dppp = 1,3-bis(diphenylphosphino)propane) complexes [27–28]. So far we have studied templates based on the Tröger’s base [27] and the 2,2’-dihydroxybinaphthyl (BINOL) [28] scaffold and identified the bend angle of these V-shaped compounds to be a critical factor for the degree of self-sorting that can be achieved [9,15–18,25–28].

The 9,9’-spirobifluorene scaffold is another very interesting concave structure that offers the possibility to orient functional groups in a defined manner [29]. Hence, we were wondering how V-shaped bis(pyridine) ligands based on this chiral skeleton would behave upon coordination to [(dppp)Pd(OTf)_2_] or [(dppp)Pt(OTf)_2_] in order to learn more about the underlying principles of chiral self-sorting in metallosupramolecular chemistry.

## Results and Discussion

### Synthesis

**Scheme 1:**
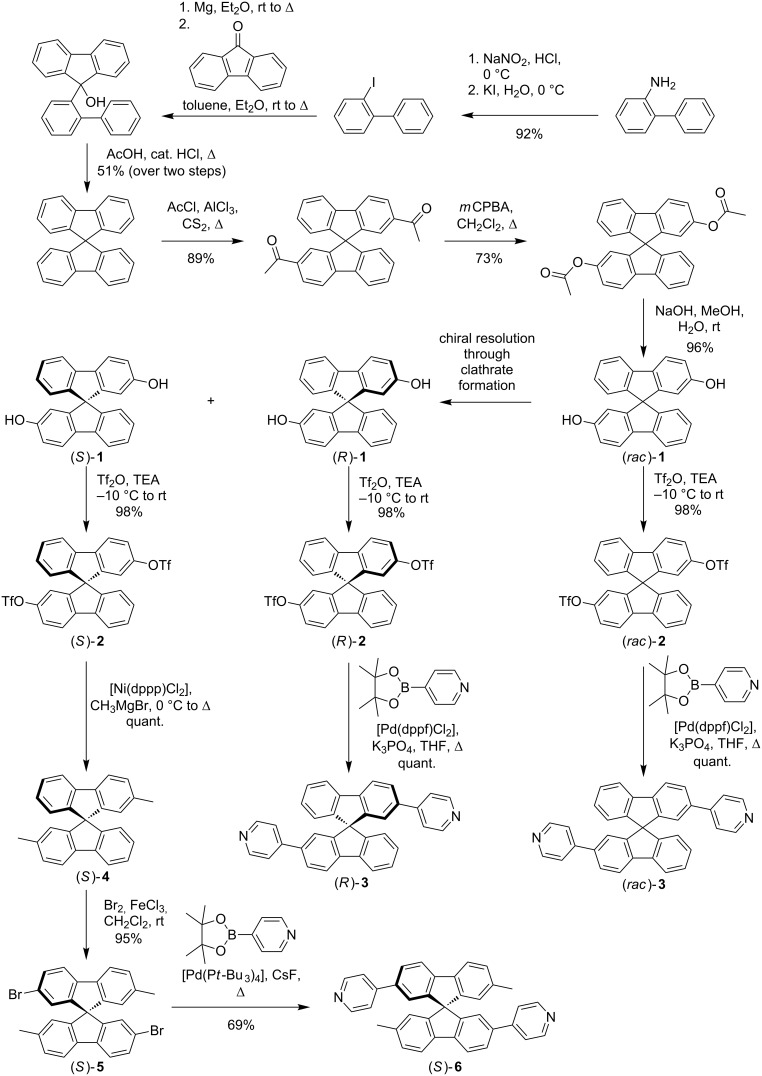
Synthesis of ligands **3** and **6**.

The synthesis of the spirobifluorene-based templates is shown in Scheme 1. It started from 2-aminobiphenyl following the established procedures of J. M. Tour [30], M. Gomberg [31], and V. Prelog [32–33] leading to (*rac*)-2,2’-dihydroxy-9,9’-spirobifluorene ((*rac*)-**1**) in six consecutive steps. This sequence involved a Sandmeyer-like iodination, followed by a Grignard reaction with fluorenone to furnish the corresponding tertiary alcohol. This alcohol was subjected to an acid-mediated condensation to give the 9,9’-spirobifluorene. Friedel–Crafts acylation with acetyl chloride gave rise to the racemic 2,2’-diketone which was transformed to the racemic diester in a Baeyer–Villiger oxidation. Saponification of the ester functions then afforded (*rac*)-**1**. One part of the racemic material was transformed into the corresponding racemic ditriflate (*rac*)-**2** which was reacted with a 4-pyridylboronic acid ester in a Suzuki cross-coupling to afford the desired racemic bis(pyridine) ligand (*rac*)-**3**. (Please note that (*rac*)-**3** has been prepared before, however, on a different way [34].)

The other part of the racemic diol (*rac*)-**1** was resolved via clathrate formation with (*R*,*R*)-2,3-dimethoxy-*N*,*N*,*N*',*N*'-tetracyclohexylsuccindiamide to obtain enantiomerically pure (*R*)-**1** and (*S*)-**1** [35–36] which were subsequently converted into the optically pure ditriflates (*R*)-**2** and (*S*)-**2**. (*R*)-**2** was then subjected to a two-fold Suzuki reaction to obtain the enantiomerically pure bis(pyridine) ligand (*R*)-**3**. (Please note, that the conditions used to elaborate the enantiomerically pure 9,9’-spirobifluorene derivatives were demonstrated not to affect the optical purity of these compounds in our previous work (see [35]).)

(*S*)-**1**, however, was reacted in a nickel-catalysed Kumada cross-coupling reaction to furnish 2,2’-dimethylated spirobifluorene (*S*)-**4** which was then brominated twice to afford tetrafunctionalised spirobifluorene (*S*)-**5**. Finally, (*S*)-**5** could be transformed into the second bis(pyridine) ligand (*S*)-**6** in a two-fold Suzuki reaction.

### Metal coordination

After the successful synthesis we mixed solutions of ligands **3** and **6** and [(dppp)Pd(OTf)_2_] or [(dppp)Pt(OTf)_2_] and characterised the resulting complexes by ^1^H, ^31^P, ^1^H 2D-DOSY NMR, ESI-MS, and single crystal X-ray diffraction to investigate if the desired rhomboid complexes are formed and whether we can observe any degree of self-sorting in the self-assembly processes of the racemic samples.

**Figure 1:**
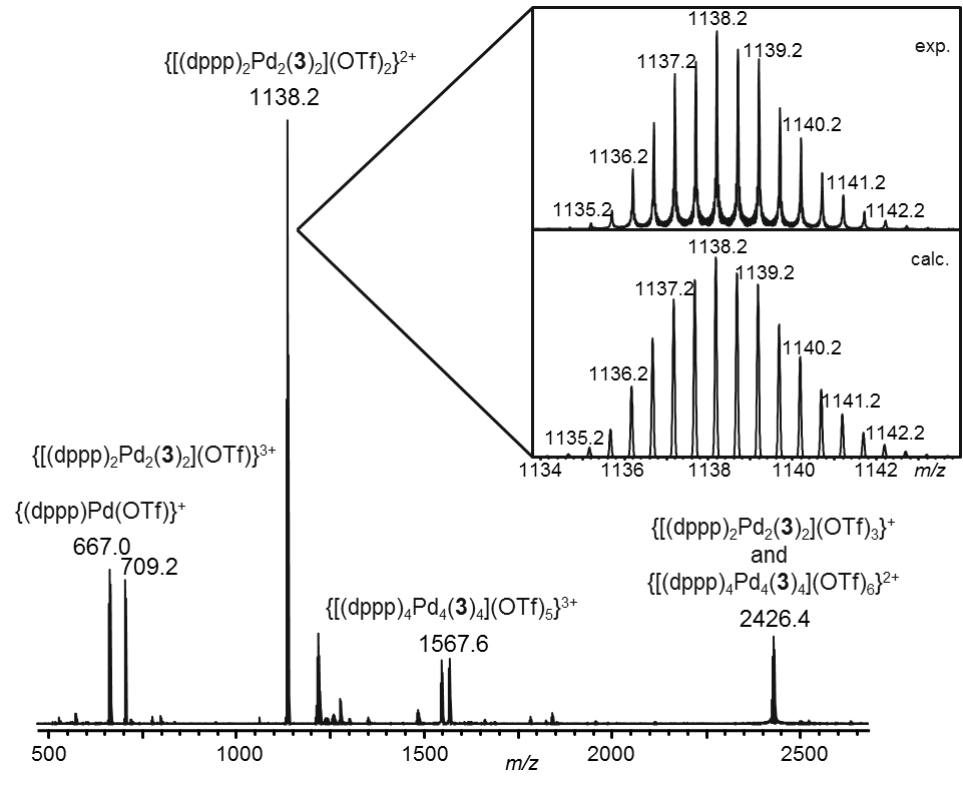
ESI mass spectrum (positive mode) of an 1:1 mixture of (*R*)-**3** and [(dppp)Pd(OTf)_2_] in acetone.

The ESI-MS spectra recorded from the complex solutions showed the successful formation of the expected dinuclear [(dppp)_2_M_2_(L)_2_] aggregates with different numbers of counter ions both from the enantiomerically pure (*R*)-**3** and the racemic ligand (*rac*)-**3** and the enantiomerically pure template (*S*)-**6**, and hence, proved the selectivity of the self-assembly processes in terms of aggregate stoichiometry (Figure 1 and Supporting Information File 1). (Please note, that we investigated our complexes in a number of different solvents (acetone, acetonitrile, mixtures of dichloromethane and acetonitrile) and in all cases we observed the expected dinuclear [(dppp)_2_M_2_(L)_2_]-complexes as the dominating species.)

**Figure 2:**
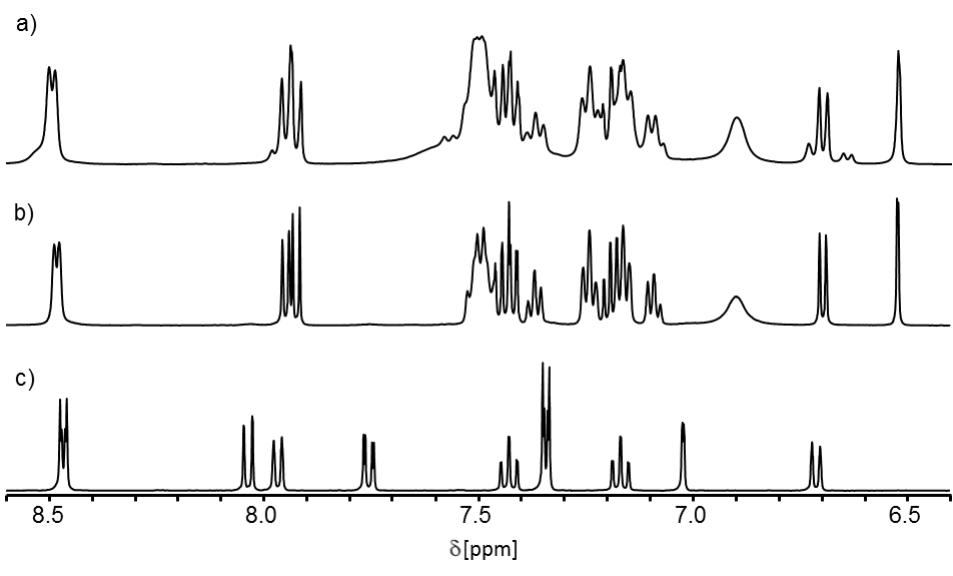
^1^H NMR spectra (500.1 MHz, in CD_2_Cl_2_/CD_3_CN 3:1 at 298 K) of a) a 1:1 mixture of [Pd(dppp)]OTf_2_ and (*rac*)-**3**, b) a 1:1 mixture of [Pd(dppp)]OTf_2_ and (*R*)-**3**, and c) (*R*)-**3**.

Next we measured ^1^H NMR spectra of the aggregates and the free ligands. All complexes of the enantiomerically pure ligands gave rise to NMR spectra with sharp but significantly shifted signals compared to the ones of the free ligands, which supports the formation of discrete metallosupramolecular species (Figure 2b,c). This could be further confirmed by 2D ^1^H DOSY NMR spectra (see Supporting Information File 1) of the aggregates as well as of the free ligands. In all cases the relative size of the aggregates was a little more than twice as large as that of the respective concave template. (Please note, that we could only determine relative sizes or ratios of sizes from the DOSY experiments because the spectra were recorded in a 3:1 mixture of dichloromethane and acetonitrile of unknown viscosity which does not allowed us to calculate hydrodynamic radii.) Therefore, these NMR-spectroscopic results nicely complement the results from mass spectrometry with regard to the assemblies’ composition.

Comparison of the ^1^H NMR spectra of the homochiral complexes of (*R*)-**3** with the spectra of the complexes obtained from the racemic ligand (*rac*)-**3** then allowed us to obtain information about the stereoselectivity of the self-assembly processes (Figure 2a,b). In principle, three stereoisomers of the dinuclear metallosupramolecular rhombi can form from racemic ligands upon coordination to [(dppp)Pd(OTf)_2_] or [(dppp)Pt(OTf)_2_] – a racemic mixture of two enantiomeric homochiral assemblies and an achiral heterochiral complex. These are expected to form in a 1:1:2 ratio if the assembly would occur in a completely non-selective purely statistical manner. Any deviation from this ratio would either mean an amplification of the racemic homochiral assemblies in the sense of self-recognition or an amplification of the heterochiral assembly in the sense of self-discrimination.

Analysis of the ^1^H NMR spectra provides versatile information: first, the self-assembly is not completely diastereoselective, since a number of additional signals are visible in the spectrum of the aggregates obtained from (*rac*)-**3**. Second, the self-assembly does not happen in a completely statistical fashion but rather proceeds with a preference for forming homochiral complexes for both palladium (Figure 2a,b) and platinum ions since the intensity of the signals of the homochiral assemblies is larger than expected from statistics. However, both diastereomers were found to give very similar NMR spectra resulting in strong signal overlap. Hence, we wanted to obtain further proof by recording ^31^P NMR spectra in which signal overlap should be considerably less problematic due to the much broader spectral width.

**Figure 3:**
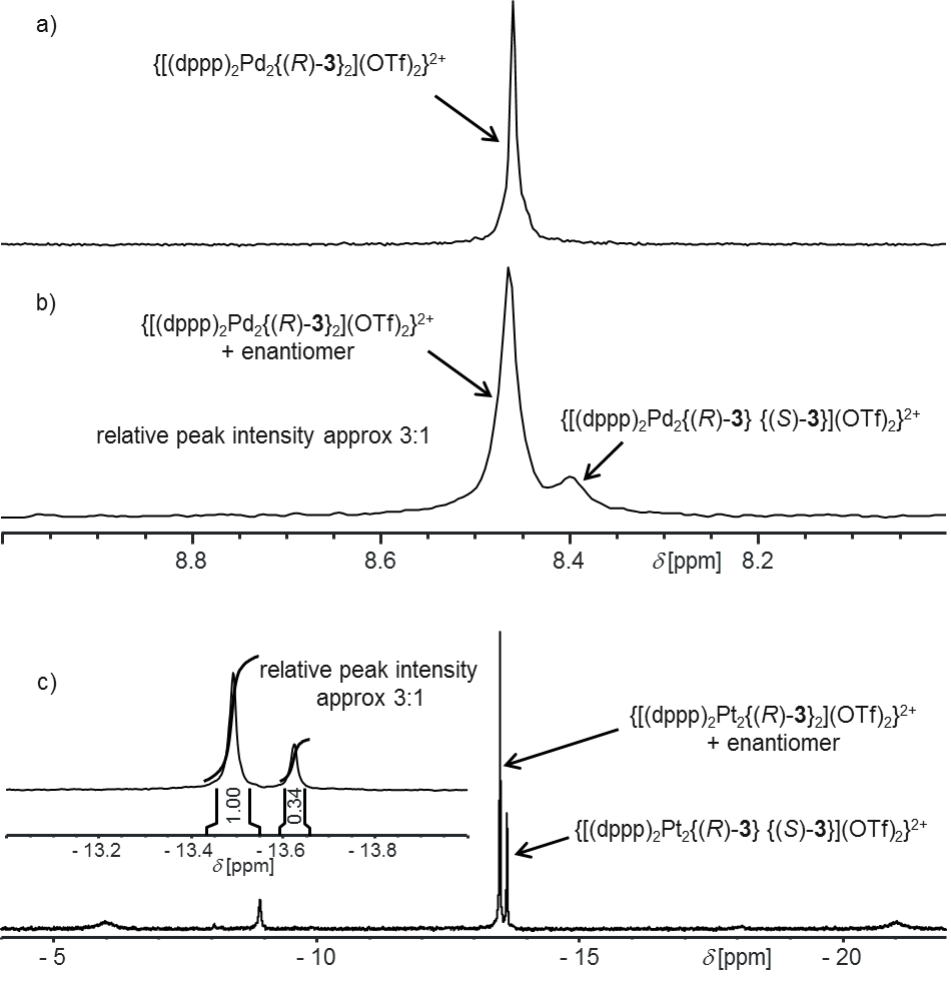
^31^P NMR spectra of a) a 1:1 mixture of [Pd(dppp)]OTf_2_ and (*R*)-**3**, b) a 1:1 mixture of [Pd(dppp)]OTf_2_ and (*rac*)-**3** (both recorded at 162.0 MHz in CD_2_Cl_2_/CD_3_CN 3:1 at 293 K), and c) of a 1:1 mixture of [Pt(dppp)]OTf_2_ and (*rac*)-**3** (202.5 MHz, in CD_2_Cl_2_ at 298 K).

As shown in Figure 3 the ^31^P NMR spectra indeed confirm the conclusions drawn from the ^1^H NMR spectra. Two individual peaks for the two diastereomers can be detected when (*rac*)-**3** is employed for complexation and the intensities of the signals of the racemic homochiral diastereomer are approximately three times as large as those of the signals of the heterochiral one in both cases – the palladium and the platinum complexes and independent of the solvents we used. This corresponds to an amplification of the homochiral stereoisomers via self-recognition by a factor of approximately three.

**Figure 4:**
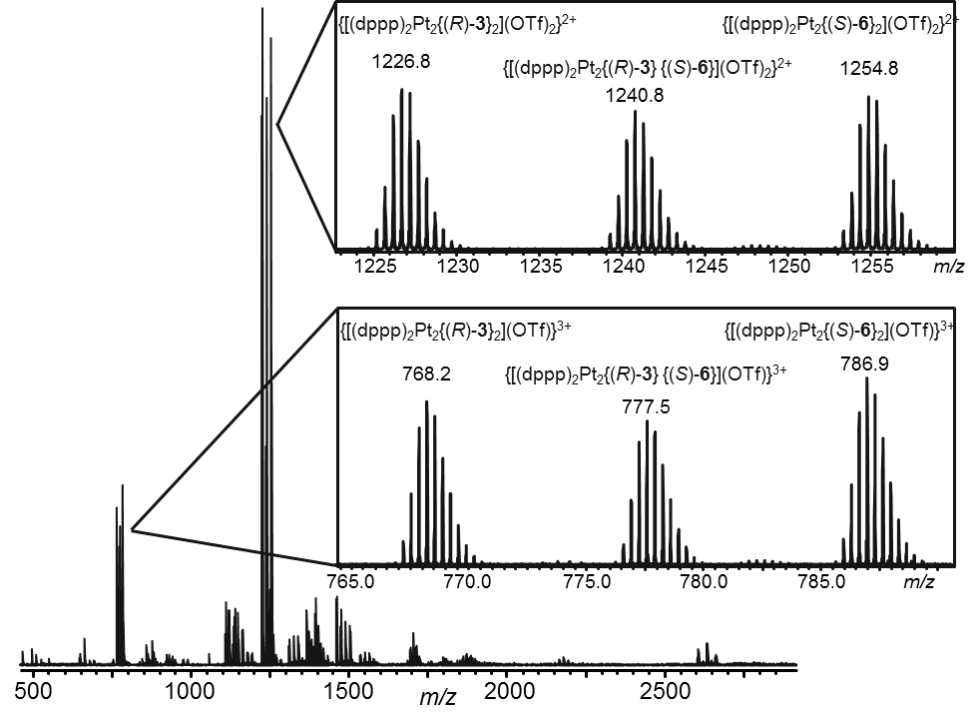
ESI mass spectrum (positive mode) of an 1:1:2 mixture of (*R*)-**3**, (*S*)-**6**, and [(dppp)Pt(OTf)_2_] in acetonitrile.

Although these NMR spectra already provide a very good proof for the stereoselectivity of the self-assembly process we wanted to get additional independent evidence by another analytical method. Standard mass spectrometry, however, cannot distinguish between different stereoisomers. In order to employ this technique for this purpose, we therefore used a 1:1 mixture of ligands (*R*)-**3** and (*S*)-**6** as a *pseudo*-racemate in which the two methyl groups of **6** in the outer periphery of the ligand structure serve as a kind of mass label. These labels then allowed us to identify the three possible different complexes due to their different number of methyl groups, and hence, different *m*/*z* values. Equimolar amounts of the enantiomerically pure ligands (*R*)-**3** and (*S*)-**6** and two equivalents of the respective metal corner were mixed. The ESI mass spectrum of the diluted solution (Figure 4) nicely corroborates the results obtained by the NMR experiments as it shows the presence of ions derived from all three complexes [(dppp)_2_M_2_{(*R*)-**3**}_2_](OTf)_4_, [(dppp)_2_M_2_{(*R*)-**3**}{(*S*)-**6**}](OTf)_4_, and [(dppp)_2_M_2_{(*S*)-**6**}_2_](OTf)_4_. Furthermore the peak ratios of the signals of these ions differ from the statistically expected 1:2:1 ratio*.* Although these peak ratios suggest a slightly lower amplification of the homochiral assemblies by a factor of only two to three, this still reinforces our analysis – especially, if one takes into account that mass labelling in the periphery might cause differences in the ESI response factors that can cause slight deviations in the relative quantification of different components compared to the ratios obtained by other techniques, e.g. NMR spectroscopy, as we have recently reported [28].

**Figure 5:**
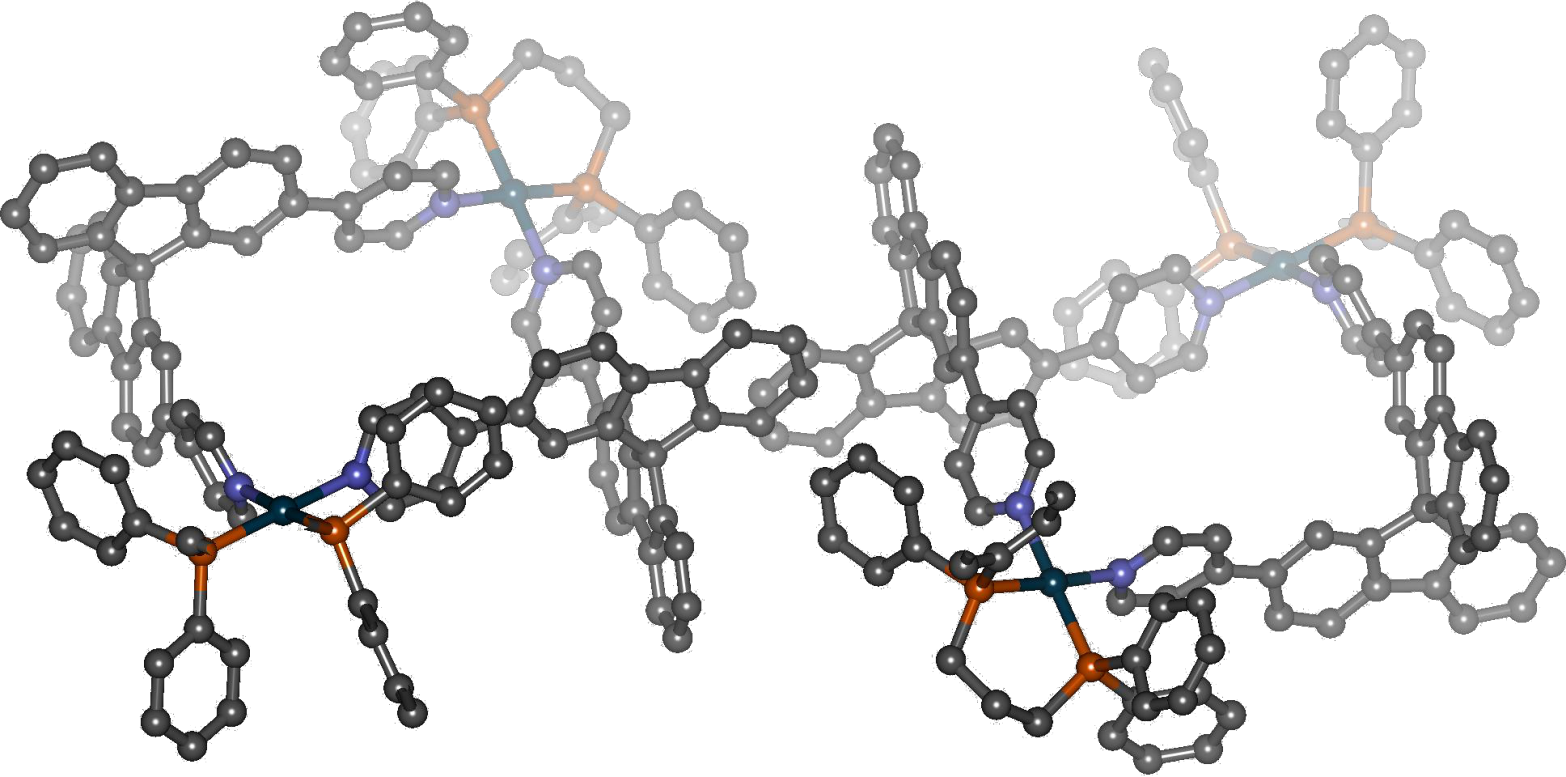
Single crystal X-ray structure analysis of [(dppp)_2_Pd_2_{(*R*)-**3**}_2_][(dppp)_2_Pd_2_{(*S*)-**3**}_2_](OTf)_8_ obtained from a 1:1 mixture of (*rac*)-**3** and [(dppp)Pd(OTf)_2_]. (Counterions and solvent molecules within the crystal packing, and hydrogen atoms are omitted, colour code: petrol – palladium, grey – carbon, blue – nitrogen, orange – phosphorous).

Finally, we were able to grow single crystals of the dinuclear palladium complexes derived from the racemic ligands from a 3:1 mixture of dichloromethane and acetonitrile using ethyl acetate as anti-solvent and characterise them by X-ray diffraction. Interestingly, we only obtained single crystals of the racemic mixture of homochiral complexes although the solution obviously contains both diastereomers. Figure 5 shows both of the homochiral complexes {[(dppp)_2_Pd_2_{(*R*)-**3**}_2_](OTf)_4_ and {[(dppp)_2_Pd_2_{(*S*)-**3**}_2_](OTf)_4_ as they were found in the crystal structure. Again, this indicates the preferred formation of the homochiral complexes in the sense of a self-recognition because in most of the cases where both the achiral heterochiral and the chiral homochiral assemblies are present in solution the heterochiral aggregates tend to crystallise more readily. (The reason for the usually preferred crystallization of the achiral assembly (compared to the corresponding diastereomeric homochiral assembly) is not yet completely clear. It might be due to the fact that nature often tries to achieve the highest possible symmetry in crystalline matter. There is also some evidence that in most cases the achiral assemblies can pack more efficiently in the solid state than the chiral ones.) Hence, selective crystallisation of the homochiral aggregate can be interpreted as another hint for its thermodynamic more favourable formation. It is important to note, however, that dissolving the crystals again very quickly led to a mixture of homo- and heterochiral assemblies in the same ratio as observed before.

## Conclusion

In summary, we were able to synthesise dissymmetric bis(4-pyridyl) ligands (*R*)- and (*rac*)-**3** based on the 9,9'-spirobifluorene scaffold in racemic and optically pure form and a closely related mass-labelled dimethylated derivative (*S*)-**6**. These ligands self-assemble into dinuclear metallosupramolecular [(dppp)_2_M_2_(L)_2_](OTf)_4_ rhombi upon coordination to [(dppp)Pt(OTf)_2_] or [(dppp)Pd(OTf)_2_], respectively. Interestingly, these self-assembly processes proceed with significant diastereoselective self-sorting via preferred narcissistic self-recognition amplifying the formation of the homochiral assemblies by a factor of approximately three. Together with the results obtained for further bis(pyridine) ligands derived from other rigid scaffolds such as Tröger's bases or 2,2'-dihydroxy-1,1'-binaphthyls this suggests that the bend angle of the V-shaped ligands seems to be a crucial general factor that has to be adjusted carefully to obtain (high-fidelity) self-sorting. Angles of more than 105° cause an unfavourable preorganisation of the ligands’ structure thus leading to non-selective self-assembly processes whereas smaller angles of about 90–105° lead to chiral self-sorting. The current study of the ligands **3** and **6** provides further evidence that angles at the lower end of this range do lead to a preferred self-recognition.

## Experimental

Reactions under inert gas atmosphere were performed under argon using standard Schlenk techniques and oven-dried glassware prior to use. Thin-layer chromatography was performed on aluminum TLC plates silica gel 60F_254_ from Merck. Detection was carried out under UV light (254 and 366 nm). Products were purified by column chromatography on silica gel 60 (70–230 mesh) from Merck. The ^1^H and ^13^C NMR spectra were recorded on a Bruker Avance 500 spectrometer at 298 K, at 500.1 and 125.8 MHz, or a Bruker AM 400 at 293 K, at 400.1 MHz and 100.6 MHz, respectively. ^31^P NMR spectra were recorded at 293 K at 162.0 MHz or at 298 K at 202.0 MHz. The ^1^H NMR chemical shifts are reported on the δ scale (ppm) relative to residual non-deuterated solvent as the internal standard. The ^13^C NMR chemical shifts are reported on the δ scale (ppm) relative to deuterated solvent as the internal standard. ^31^P NMR chemical shifts are reported on the δ scale (ppm) relative to 85% H_3_PO_4_ as the external standard. Signals were assigned on the basis of ^1^H, ^13^C, HMQC, and HMBC NMR experiments (for the numbering of the individual nuclei see Supporting Information). Mass spectra were recorded at a microOTOF-Q or an Apex IV FT-ICR from Bruker. Most solvents were dried, distilled, and stored under argon according to standard procedures. 4-Pyridylboronic acid pinacol ester and 2-aminobiphenyl were used as received from commercial sources. 2-Iodobiphenyl [30], 9,9'-spirobifluorene [31], (*rac*)-2,2'-diacetyl-9,9'-spirobifluorene [32], (*rac*)-2,2'-diacetoxy-9,9'-spirobifluorene [33], (*rac*)-, (*S*)-, and (*R*)-2,2'-dihydroxy-9,9'-spirobifluorene {(*rac*)-, (*S*)-, and (*R*)-**1**} [33,36], (*rac*)-, (*S*)-, and (*R*)-2,2'-bis(trifluoromethylsulfonyloxy)-9,9'-spirobifluorene {(*rac*)-, (*S*)-, and (*R*)-**2**} [36], (*S*)-2,2'-dimethyl-9,9'-spirobifluorene {(*S*)-**4**} [36], (*S*)-2,2'-dibromo-7,7'-dimethyl-9,9'-spirobifluorene {(*S*)-**5**} [36], and (dppp)M(OTf)_2_ (M = Pd^II^, Pt^II^) [37–38] were prepared according to literature known procedures.

**(*R*)-2,2’-Bis(4-pyridyl)-9,9’-spirobifluorene ((*R*)-3):** (*R*)-**2** (339 mg, 0.55 mmol), K_3_PO_4_ (476 mg, 2.24 mmol), 4-pyridylboronic acid pinacol ester (551 mg, 2.69 mmol), [Pd(dppf)Cl_2_] (25 mg, 0.031 mmol), and dppf (22 mg, 0.040 mmol) were added to a round-bottomed flask. Dry THF (10 mL) was added and the mixture was heated to 65 °C and stirred overnight resulting in a brown solution. After that the reaction mixture was cooled to rt and quenched with a sat. solution of NaHCO_3_. The aqueous phase was extracted three times with dichloromethane and the collected organic phase was washed with water and brine and dried with MgSO_4_. The solvent was removed in vacuo. The crude product was purified by column chromatography on silica gel using MeOH/ethyl acetate (1:1) as eluent. The solvents were evaporated and the residue extracted with dichloromethane to get rid of previously dissolved silica gel. After evaporation of the dichloromethane the product was obtained as a brown oil (262 mg, 100%). ^1^H NMR (500.1 MHz, CDCl_3_) δ 8.50 (d, *J*_15,16_ = 5.4 Hz, 4H, H-16), 7.97 (d, *J*_3,4_ = 7.9 Hz, 2H, H-4), 7.91 (d, *J*_5,6_ = 7.5 Hz, 2H, H-5), 7.68 (dd, *J*_3,4_ = 7.9 Hz, *J*_1,3_ = 1.0 Hz, 2H, H-3), 7.41 (dd, *J*_5,6_ = 7.5 Hz, *J*_6,7_ = 7.3 Hz, 2H, H-6), 7.33 (d, *J*_15,16_ = 5.4 Hz, 4H, H-15), 7.15 (dd, *J*_6,7_ = 7.3 Hz, *J*_7,8_ = 7.5 Hz, 2H, H-7), 7.00 (s, 2H, H-1), 6.77 (d, *J*_7,8_ = 7.5 Hz, 2H, H-8) ppm; ^13^C NMR (125.8 MHz, CDCl_3_) δ 150.0 (C-16), 149.4 (C-10), 148.9 (C-13), 148.0 (C-14), 142.9 (C-11), 140.9 (C-12), 137.8 (C-2), 128.6 (C-7), 128.2 (C-6), 127.1 (C-3), 124.2 (C-8), 122.5 (C-1), 121.5 (C-14), 120.8 (C-4), 120.5 (C-5), 66.0 (C-9) ppm; HRMS-ESI (*m*/*z*): [M + H]^+^ calcd for [C_35_H_23_N_2_]^+^, 471.1856; found, 471.1861; [α]_D_^25^ +280.7° (*c* 0.385, CHCl_3_).

**(*S*)-2,2'-Bis(4-pyridyl)-7,7'-dimethyl-9,9'-spirobifluorene ((*S*)-6):** CsF (602 mg, 3.96 mmol) was dried under vacuum. After flushing with argon (*S*)-**5** (300 mg, 0.60 mmol), 4-pyridylboronic acid pinacol ester (148 mg, 0.72 mmol), and [Pd(P*t-*Bu_3_)_2_] (15 mg, 5 mol %) were added and the reaction flask was evacuated twice. Next dry THF (10 mL) was added and the reaction was heated to 65 °C overnight. After that the reaction mixture was cooled to rt and diluted with dichloromethane. The organic phase was washed with a saturated solution of Na_2_CO_3_ and the aqueous phase was repeatedly extracted with dichloromethane. The collected organic phases were washed with a diluted hydrochloric acid (5%) and the resulting aqueous phase was extracted thoroughly with CH_2_Cl_2_. The aqueous phase was brought to pH 14 with 2 N NaOH and extracted thoroughly with CH_2_Cl_2_. The collected organic phases were washed with water and brine and subsequently dried with MgSO_4_. The solvents were removed in vacuo. The crude product was purified by column chromatography on silica gel using petrol ether/ethyl acetate/triethylamine (5:1:1) as eluent resulting in a yellow solid (207 mg, 69%). ^1^H NMR (500.1 MHz, CDCl_3_) δ 8.50 (d, *J*_15,16_ = 5.6 Hz, 4H, H-16), 7.91 (d, *J*_3,4_ = 7.9 Hz, 2H, H-4), 7.78 (d, *J*_6,5_ = 7.8 Hz, 2H, H-6), 7.67 (dd, *J*_3,4_ = 7.9 Hz, *J*_1,3_ = 1.6 Hz, 2H, H-3), 7.33 (m, 4H, H-15), 7.22 (d, *J*_5,6_ = 7.8 Hz, 2H, H-6), 6.99–6.98 (d, *J*_1,3_ = 1.6 Hz, 2H, H-1), 6.56 (s, 2H, H-8), 2.23 (s, 6H, H-17) ppm; ^13^C NMR (125.8 MHz, CDCl_3_) δ 149.7 (C-16)*, 149.5 (C-10)*, 149.2 (C-13), 147.9 (C-14), 143.0 (C-11), 138.6 (C-7), 138.2 (C-12), 137.1 (C-2), 129.0 (C-6), 126.9 (C-3), 124.7 (C-8), 122.4 (C-1), 121.3 (C-15), 120.3 (C-5)*, 120.2 (C-4)*, 65.7 (C-9), 24.8 (C-17) ppm (* signal assignment might be interchanged); HRMS-ESI (*m*/*z*): [M + H]^+^ calcd for [C_37_H_27_N_2_]^+^, 499.2169; found, 499.2160. [α]_D_^24^ −128.1° (*c* 0.39, CHCl_3_).

**Preparation and characterisation of the metal complexes:** Approximately 10 µmol of a ligand and an equimolar amount of [(dppp)Pt(OTf)_2_] or [(dppp)Pd(OTf)_2_], respectively, were dissolved in 0.6 mL of CD_2_Cl_2_ and 0.2 mL of CD_3_CN. The resulting solution was characterised by NMR. For the ESI-MS studies ca. 10 µmol of a ligand and an equimolar amount of [(dppp)Pt(OTf)_2_] or [(dppp)Pd(OTf)_2_], respectively, were dissolved in 1 mL of solvent (acetone, acetonitrile, mixtures of dichloromethane and acetonitrile). An aliquot of this solution was then diluted with the same solvent to give 1 mL of a 300 µmolar solution of the complex.

**[(dppp)_2_Pd_2_{(*R*)-3}_2_](OTf)_4_: **^1^H NMR (500 MHz, CD_3_CN/CD_2_Cl_2_ 1:1) δ 8.48 (d, *J*_15,16_ = 5.8 Hz, 4H, H-16), 7.94 (d, *J*_5,6_ = 7.7 Hz, 2H, H-5), 7.92 (d, *J*_3,4_ = 8.0 Hz, 2H, H-4), 7.53–7.46 (m, 8H, H-dppp), 7.44 (dd, *J*_5,6_ = 7.7 Hz, *J*_6,7_ = 7.7 Hz, 2H, H-6), 7.41 (dd, *J*_3,4_ = 8.1 Hz, *J*_1,3_ = 1.5 Hz, 2H, H-3), 7.36 (dd, *J* = 7.5 Hz, *J* = 7.5 Hz, 2H, H-dppp), 7.24 (dd, *J* = 7.4 Hz, 4H, H-dppp), 7.21–7.14 (m, 6H, H-7, H-dppp), 7.09 (dd, *J* = 7.3 Hz, *J* = 7.3 Hz, 2H, H-dppp), 6.90 (s, 6H, H15), 6.69 (dd, J_7,8_ = 7.5 Hz, 2H, H-8), 6.52 (d, *J*_1,3_ = 1.5 Hz, 2H, H-1), 3.10–3.00 (m, 4H, H-dppp), 2.23–2.06 (m, 2H, H-dppp); ^13^C NMR (125.8 MHz, CD_3_CN/CD_2_Cl_2_ 1:1) δ 150.1, 149.9, 149.5, 148.4, 144.1, 140.8, 134.8, 133.1, 132.9, 132.3, 132.0, 129.4, 129.1, 128.5, 127.1, 125.9, 125.4, 125.4, 125.0, 123.9, 123.3, 122.4, 122.1, 121.3, 121.2, 119.9, 65.7, 21.4, 17.5; MS (ESI, positive mode, acetone) *m*/*z*: 667.0 {(dppp)Pd(OTf)}^+^, 709.2 {[(dppp)_2_Pd_2_{(*R*)-**3**}_2_](OTf)}^3+^, 1138.2 {[(dppp)_2_Pd_2_{(*R*)-**3**}_2_](OTf)_2_}^2+^, 1567.6 {[(dppp)_4_Pd_4_{(*R*)-**3**}_4_](OTf)_5_}^3+^, 2426.4 {[(dppp)_2_Pd_2_{(*R*)-**3**}_2_](OTf)_3_}^+^ and {[(dppp)_4_Pd_4_{(*R*)-**3**}_4_](OTf)_6_}^2+^.

**[(dppp)_2_Pd_2_{(*S*)-6}_2_](OTf)_4_: **^1^H NMR (500.1 MHz, CD_3_CN/CD_2_Cl_2_ 1:1) δ 8.47 (d, *J*_15,16_ = 5.5 Hz, 4H, H-16), 7.86 (d, *J*_3,4_ = 7.9 Hz, 2H, H-4), 7.82 (d, *J*_5,6_ = 7.9 Hz, 2H, H-5), 7.54–7.45 (m, 9H, H-dppp), 7.39 (dd, *J*_3,4_ = 7.9 Hz, *J*_1,3_ = 1.6 Hz, 2H, H-3), 7.36 (d, *J* = 7.5 Hz, 2H, H-dppp), 7.28–7.21 (m, 6H, H-6, H-dppp), 7.16 (dd, *J* = 7.1 Hz, *J* = 7.1 Hz, 4H, H-dppp), 7.08 (dd, *J* = 7.3 Hz, *J* = 7.3 Hz, 2H, H-dppp), 6.89 (s, 3H, H-15), 6.52 (s, 2H, H-8), 6.49 (d, *J*_1,3_ =1.6 Hz, 2H, H-1), 3.09–3.01 (m, 4H, H-dppp), 2.19 (s, 6H, H-17); ^13^C NMR (125.8 MHz, CD_3_CN/CD_2_Cl_2_ 1:1) δ 150.1, 149.9, 149.6, 148.8, 144.3, 139.4, 138.1, 134.2, 133.2, 133.0, 132.3, 131.9, 129.4, 126.0, 125.9, 125.5, 125.4, 125.0, 124.5, 123.3, 122.4, 122.1, 120.9, 119.9, 65.4, 21.5, 21.1, 17.5; MS (ESI, positive mode, acetone) *m*/*z*: 667.0 {(dppp)Pd(OTf)}^+^, 1166.3 {[(dppp)_2_Pd_2_{(*S*)-**6**}_2_](OTf)_2_}^2+^, 2482.5 {[(dppp)_2_Pd_2_{(*S*)-**6**}_2_](OTf)_3_}^+^ and {[(dppp)_4_Pd_4_{(*S*)-**6**}_4_](OTf)_6_}^2+^.

**Crystal structure determination:** Data for the X-ray crystallographic analysis of [(dppp)_2_Pd_2_{(*R*)-**3**}_2_][(dppp)_2_Pd_2_{(*S*)-**3**}_2_](OTf)_8_ derived from (*rac*)-**3** and [(dppp)Pd(OTf)_2_] were collected on a SuperNova Dual Source diffractometer equipped with an Atlas detector using mirror monochromated Mo K_a_ radiation (λ = 0.71073 Å). CrysAlisPro software [39] was used for data measurement and processing as well as to apply the numerical absorption correction. The structures were solved by direct methods (SIR97 [40]) and refined by full-matrix least squares on *F*^2^ (SHELXL-2013 [41]). All non-hydrogen atoms were refined anisotropically. The SQUEEZE routine of PLATON [42] was used to calculate the contributions of the disordered solvent regions to the structure factors in the form of FAB files which were then used in refinement in SHELXL-2013. The hydrogen atoms at carbon were placed in calculated positions and refined isotropically using a riding model. Selected data: Crystal dimensions 0.63 × 0.29 × 0.25 mm^3^, colourless prism, C_144_H_128_F_12_N_4_O_20_P_4_Pd_2_S_4_, *M* = 2927.42, monoclinic, space group *C* 2/*c* (no. 15), *a* = 18.8830(5), *b* = 23.7387(5), *c* = 35.9013(8) Å, α = 90, β = 101.585(2), γ = 90°, *V* = 15765.2(6) Å ^3^, *Z* = 4, ρ = 1.233 g cm^−3^, μ = 0.395 mm^−1^, F(000) = 6016, 27330 reflections (2*θ*_max_ = 50.50°) measured (14210 unique, *R*_int_ = 0.0193, completeness = 99.4%), *R* (*I* > 2*σ*(*I*)) = 0.0505, *wR*_2_ (all data) = 0.1399. GOF = 1.061 for 942 parameters and 669 restraints, largest diff. peak and hole 1.548/−0.653 e Ǻ^3^.

CCDC-971933 contains the supplementary data for this structure. These data can be obtained free of charge via http://www.ccdc.cam.ac.uk/data_request/cif, or by emailing data_request@ccdc.cam.ac.uk, or by contacting The Cambridge Crystallographic Data Centre, 12, Union Road, Cambridge CB2 1EZ, UK; fax: +44 1223 336033.

## Supporting Information

File 1 NMR and ESI mass spectra of ligands **3** and **6** and their metal complexes.
