# Supplementary material for: Self-assembly of metallosupramolecular rhombi from chiral concave 9,9’-spirobifluorene-derived bis(pyridine) ligands
Source: Beilstein J Org Chem. 2014 Feb 18;10:432–41. doi: 10.3762/bjoc.10.40 (PMC3943293; doi:10.3762/bjoc.10.40)
Supplement: File 1 — NMR and ESI mass spectra of ligands 3 and 6 and their metal complexes. [file Beilstein_J_Org_Chem-10-432-s001.pdf]

Supporting Information

for

Self-assembly of metallosupramolecular rhombi from chiral concave 9,9’-spirobifluorene-derived bis(pyridine) ligands

Rainer Hovorka[1], Sophie Hytteballe[1], Torsten Piehler[1], Georg Meyer-Eppler[1], Filip Topić[2], Kari Rissanen[2], Marianne Engeser[3] and Arne Lützen[1*]

Address: [1]University of Bonn, Kekulé-Institute of Organic Chemistry and Biochemistry, Gerhard-Domagk-Str. 1, D-53121 Bonn, Germany, [2]Department of Chemistry, Nanoscience Center, University of Jyväskylä, P.O. Box 35, 40014 Jyväskylä, Finland and [3]University of Bonn, Kekulé-Institute of Organic Chemistry and Biochemistry, Gerhard-Domagk-Str. 1, D-53121 Bonn, Germany

Email: Arne Lützen – arne.luetzen@uni-bonn.de

*Corresponding author

NMR and ESI mass spectra of compounds 3 and 6 and their metal complexes

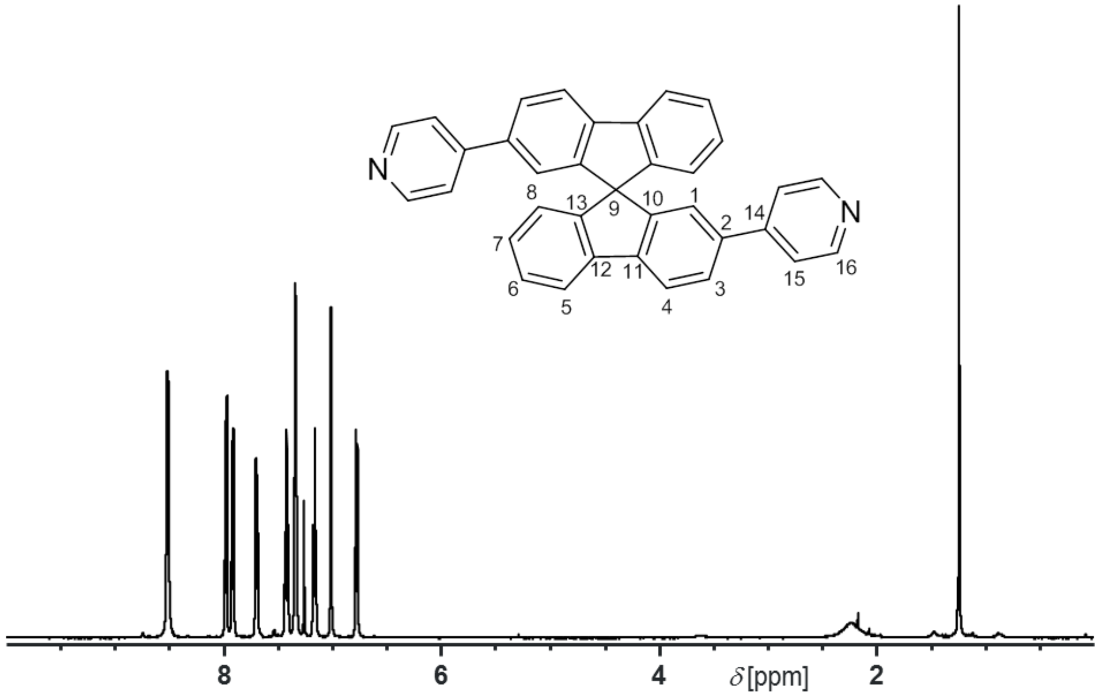

Figure S1: ^{1}H NMR (400.1 MHz, in $CDCl_3$ at 293 K) of (*rac*)-**3**.

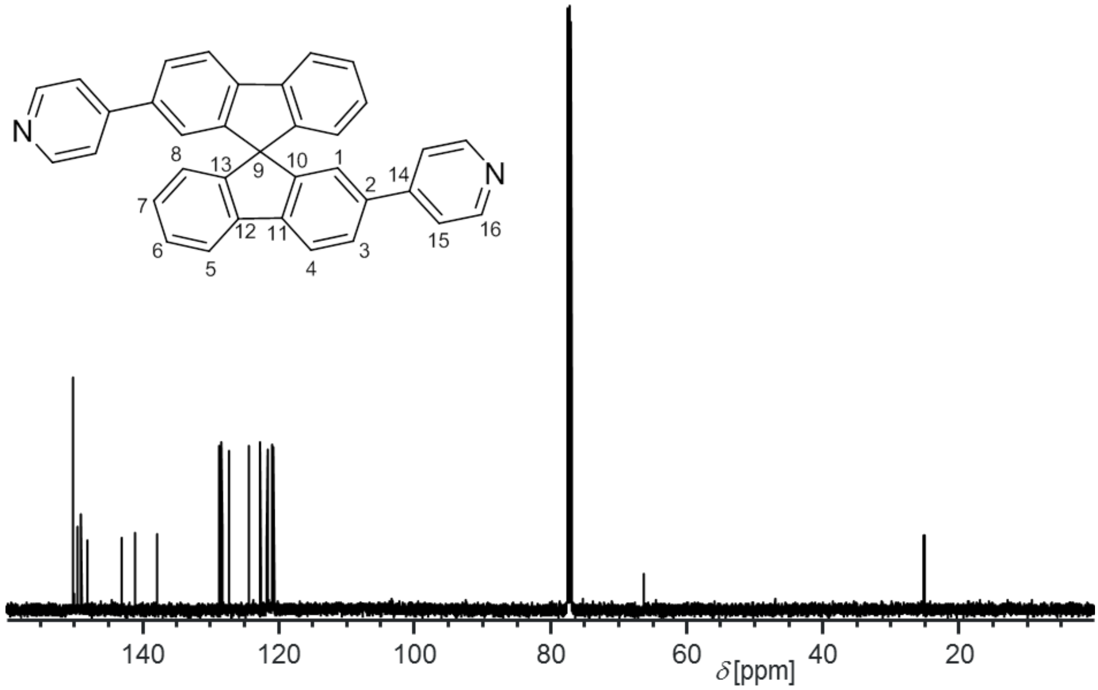

Figure S2: ^{13}C NMR (100.6 MHz, in $CDCl_3$ at 293 K) of (*rac*)-**3**.

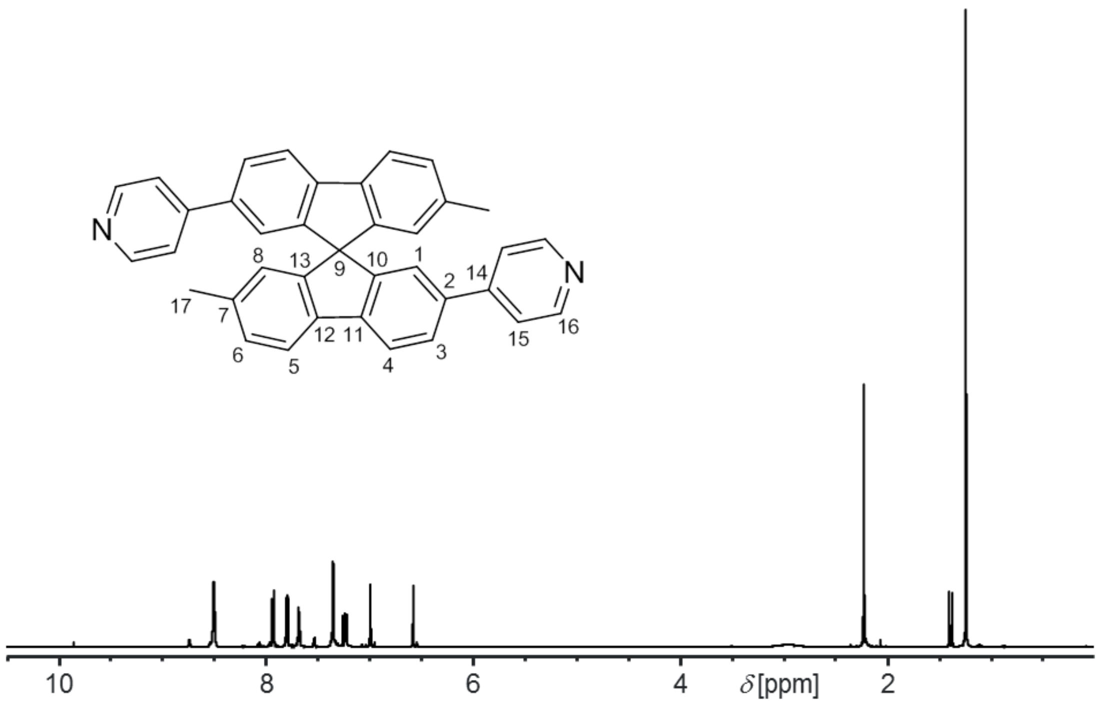

Figure S3: ^{1}H NMR (400.1 MHz, in $CDCl_3$ at 293 K) of (*S*)-**6**.

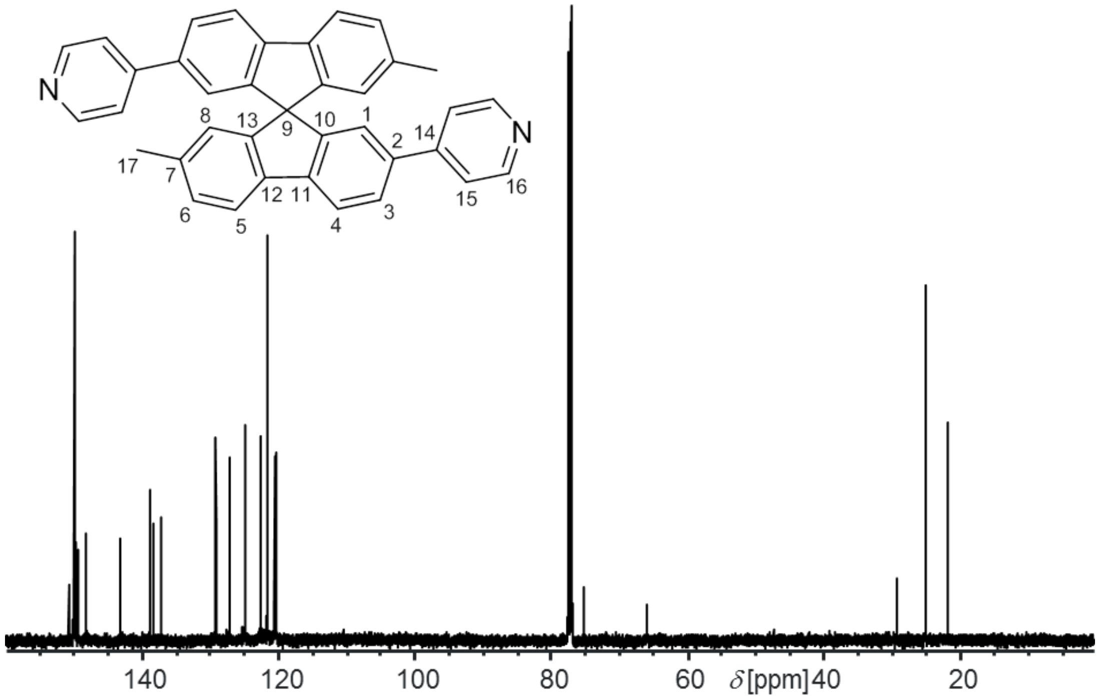

Figure S4: ^{13}C NMR (100.6 MHz, in $CDCl_3$ at 293 K) of (*S*)-**6**.

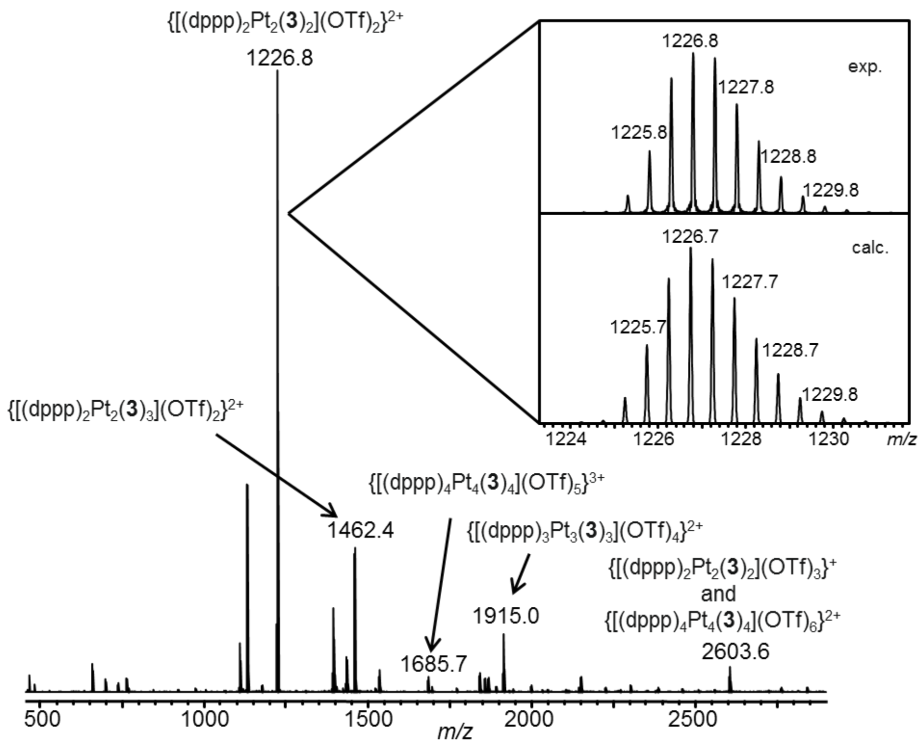

Figure S5: ESIMS (positive mode, sprayed from an acetone solution) of $[(dppp)_2Pt_2\{(R)\text{-}\mathbf{3}\}_2](OTf)_4$.

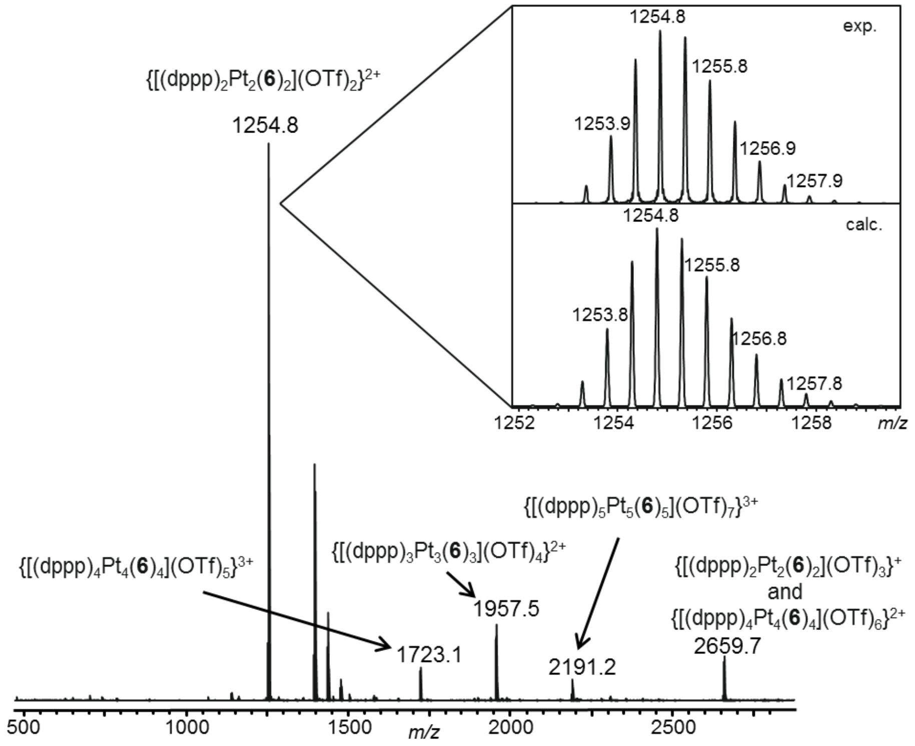

Figure S6: ESIMS (positive mode, sprayed from an acetone solution) of $[(dppp)_2Pt_2\{(S)\text{-}\mathbf{6}\}_2](OTf)_4$.

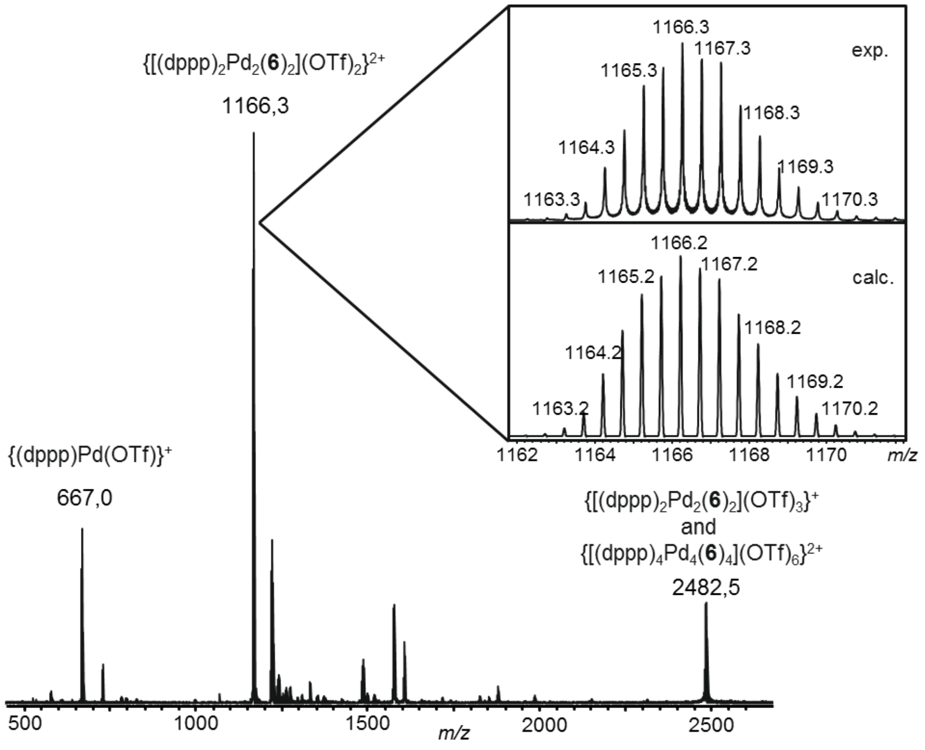

Figure S7: ESIMS (positive mode, sprayed from an acetone solution) of $[(dppp)_2Pd_2\{(S)\text{-}\mathbf{6}\}_2](OTf)_4$.

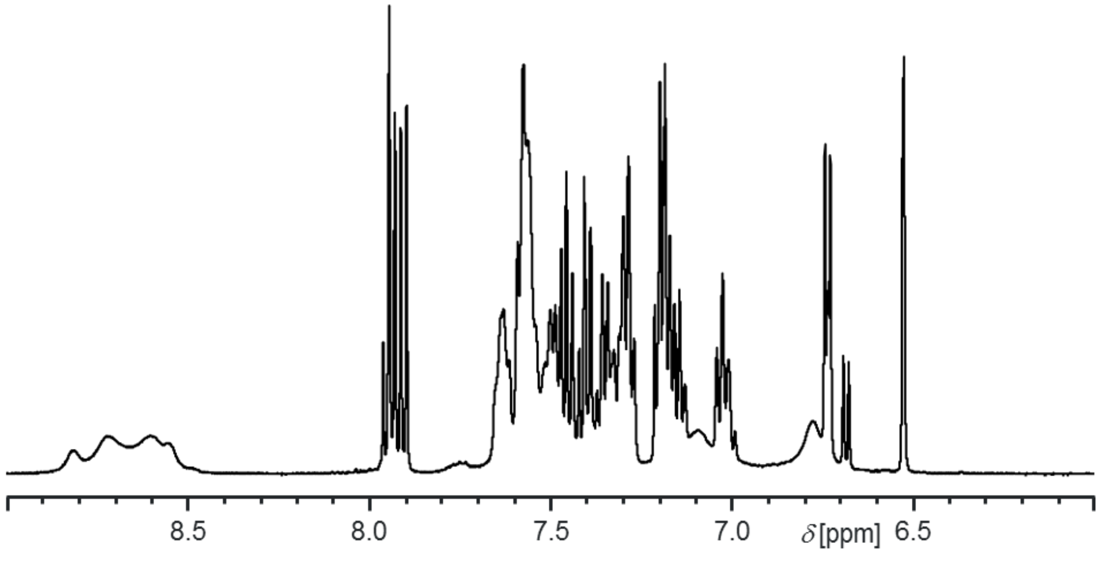

Figure S8: ^{1}H NMR (500.1 MHz, in CD_2Cl_2 at 298 K) of a 1:1 mixture of $[Pt(dppp)]OTf_2$ and (*rac*)-**3**.

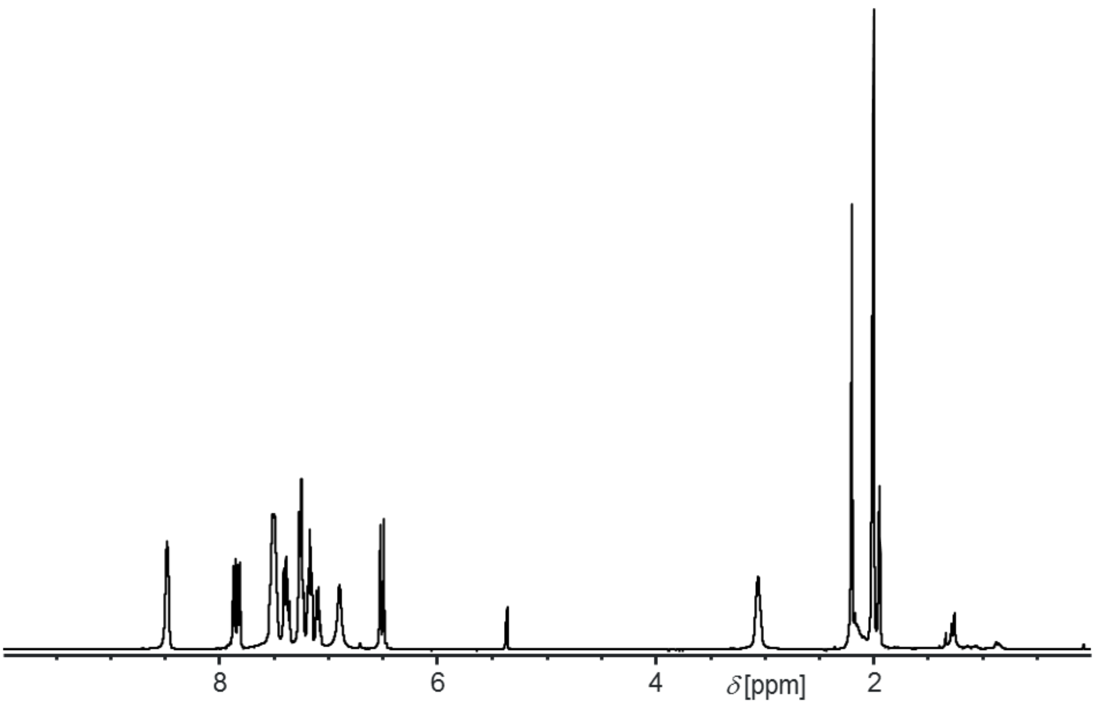

Figure S9: ^{1}H NMR (500.1 MHz, in CD_2Cl_2/CD_3CN 3:1 at 298 K) of a 1:1 mixture of [Pd(dppp)]OTf_2 and (*S*)-**6**.

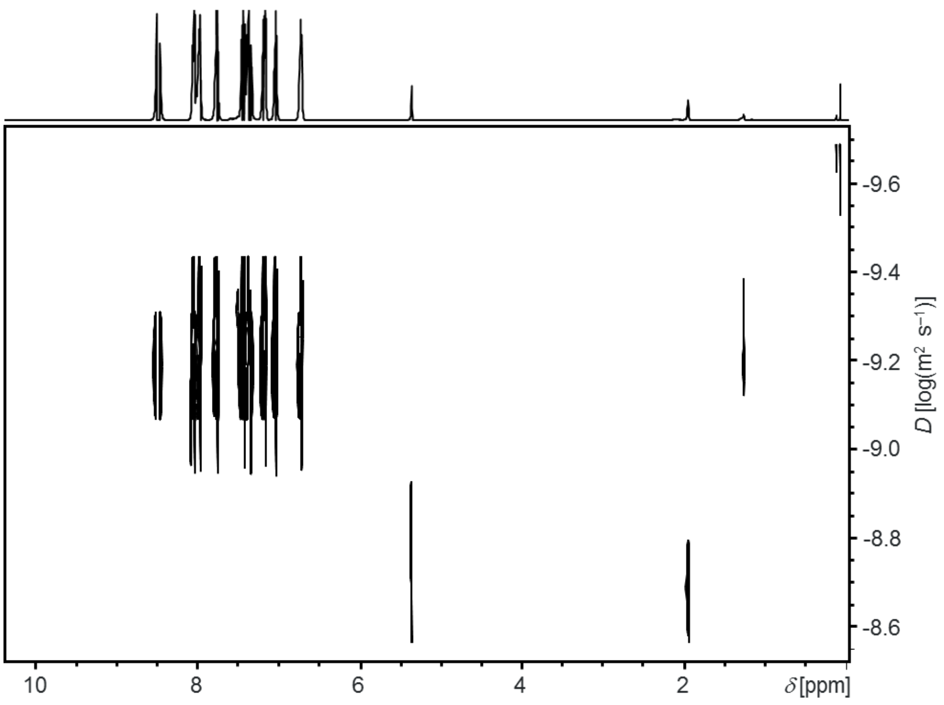

Figure S10: ^{1}H 2D-DOSY-NMR (500.1 MHz, in CD_2Cl_2/CD_3CN 3:1 at 298 K) of (*rac*)-**3**.

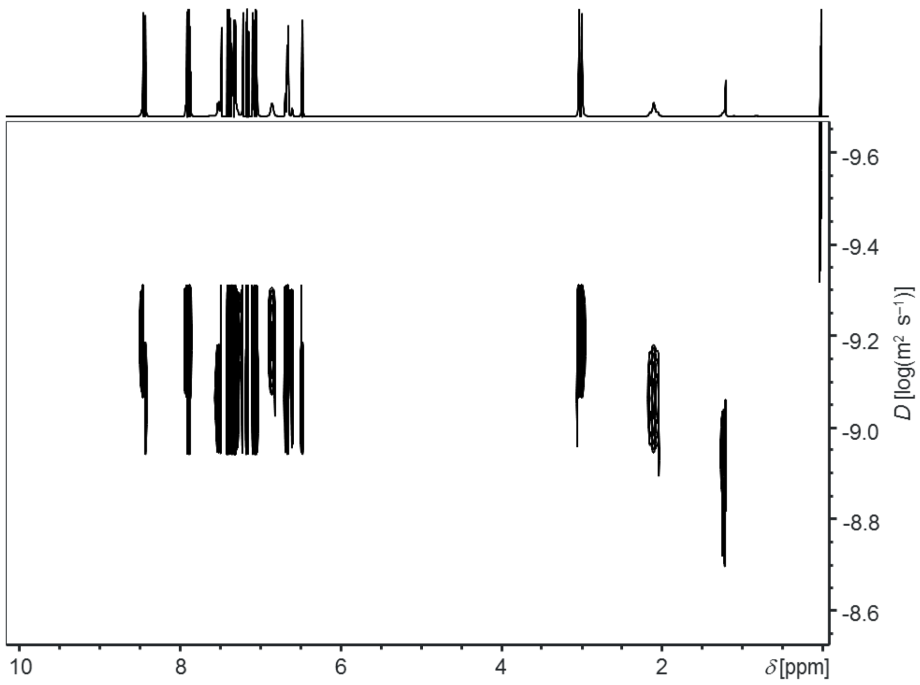

Figure S11: ^{1}H 2D-DOSY-NMR (500.1 MHz, in CD_2Cl_2/CD_3CN 3:1 at 298 K) of a 1:1 mixture of [Pd(dppp)]OTf_2 and (*rac*)-**3**.

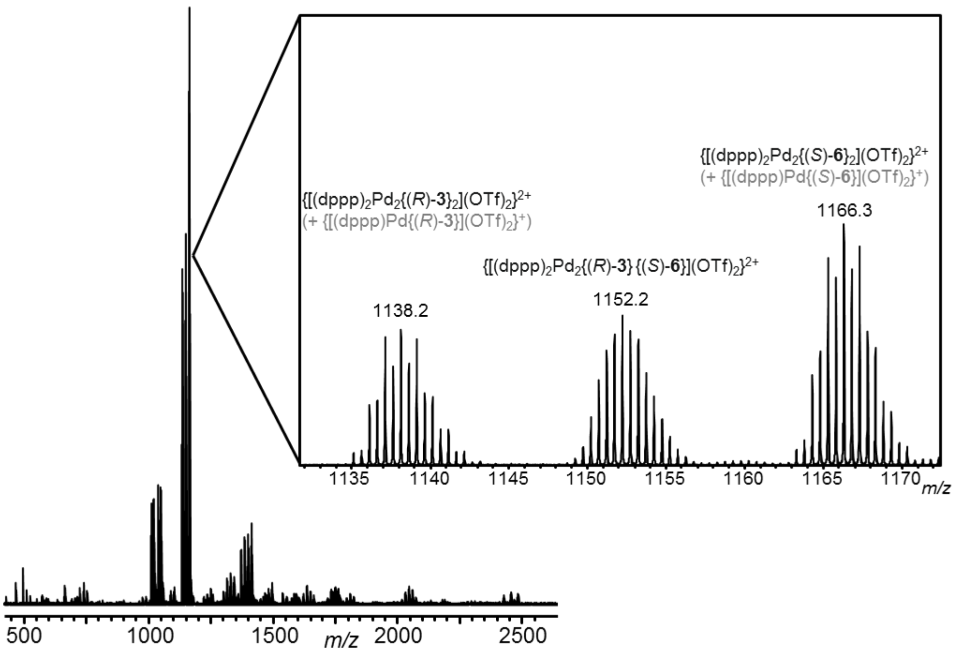

Figure S12: ESIMS (positive mode, sprayed from an acetonitrile solution) of a mixture of 2 equiv [(dppp)Pd$(OTf)_2$] + 1 equiv (*R*)-**3** + 1 equiv (*S*)-**6**.
